# Employment of the Iodide of Potassium as a Remedy for the Affections by Lead and Mercury

**Published:** 1853-05

**Authors:** 


*On the Employment of Iodide of Potassium as a Remedy for the Affections caused by Lead and Mercury.* By M. Melsens. (Translated from the paper in the "Annales de Chimie et de Physique," June, 1849.") By William Budd, M. D., Physician to the Bristol Royal Infirmary.

In the January No., 1853, of the British and Foreign Medico-Chirurgical Review, an article under the above caption appears, prefaced by some valuable remarks from the pen of Dr. William Budd, in which he sets forth the highly interesting character of the evidence by which the facts brought forward by M. Melsens are authenticated.

If, as M. Melsens alledges, and his numerous experiments seem conclusively to show, iodide of potassium is not only a safe, certain and radical cure for the common forms of saturnine and mercurial poisoning, but an equally sure preventive of the injurious effects so frequently produced by emanations from lead and mercury, the fact is one which cannot be made too widely known.

In all cases of mercurial and saturnine poisoning, he assumes that the metallic substance is in actual union with the affected parts, and is retained there in the form of some insoluble compound.

According to this view, the iodide of potassium, after its absorption into the blood, combines with the metallic poison, and forms with it a new and soluble salt—liberates the poison from its union with the injured part—dissolves it out, so to speak, from the damaged fiber, and sets it once more afloat in the circulation.

The new compound thus set at liberty, (under the form, it is presumed, of a double iodide of mercury and potassium,) he supposes to be eliminated through the kidneys almost as soon as formed, in combination with any excess of iodide of potassium that may happen to be present. So that both poison and remedy being cast out together, the cure may be said, in a peculiar sense, to be radical and complete.

Dr. Budd relates the case of a man who was admitted to the Bristol Royal Infirmary, about three years ago, for the cure of secondary syphilis, affecting his throat, joints and bones. He was also the subject of partial paralysis of the lower extremities, which had come on concurrently with the other symptoms. He had had chancre for some months before, for which, from his statement, he took mercury largely. From the time when he had chancre, (five months previously,) up to the day of his admission to the Infirmary, he had not taken any mercurial compound. The day after he came in he was put under treatment by iodide of potassium in pretty free doses. A few days afterward profuse salivation took place. The swollen face, the peculiar state of the tongue, the loosened teeth, the ulcerated mouth, and the characteristic fœtor, were all present; and yet this man, as was specially ascertained, had not taken a grain of mercury since his admission.

Under these circumstances, Dr. Budd concludes that a portion of the mercury, administered some months before, had become fixed in the body, and that the liberation of this mercury, under the solvent influence of the iodide of potassium, was the cause of the severe salivation under which the patient was now laboring. No other theory of the case, in fact, seems admissible. It may not be uninteresting to mention, that under the continuance of the iodide not only the syphilitic affection soon got better, but the paralysis, also, was almost entirely cured.

Since the occurrence of the case, Dr. Budd has seen many others of the same kind, in which mercurial ptyalism came on during the use of the iodide of potassium, in persons who had taken mercury some weeks or months before, but none within a more recent period.

M. Mulsens begins his memoir by expressing a hope that medical men will pardon him for having treated the question of the cure of the disorders produced by metallic compounds in a purely chemical point of view. "From the very outset of my researches," says he, "I have always looked at the subject in its simplest aspect. I have never, in fact, kept in view more than two definite things—*the disease from the presence of poison in the system, and the cure by the expulsion of this poison out of the system.*"

It appears from the result of many experiments, that the iodide of potassium does not tend indifferently to every part of the body, but that marked differences occur in the quantity found in different organs. Thus blood from the heart and the liver do not appear to contain similar quantities of the iodides; iodides are found in the liver when they are absolutely wanting in the liquid of the gall bladder; the serum which bathes the intestinal canal, the pleura, etc., contains compounds of iodine, but the matters found in the intestine itself contained ordinarily none beyond the first half of the gut.

The principle of the treatment by the iodide of potassium, is to render soluble any metallic compounds which have become fixed in the living body, and to facilitate their elimination by uniting them with a substance most readily cast out of the system. In a chemical point of view, this plan of treatment differs essentially from any hitherto proposed. * * * * It has been generally supposed that mercury and lead are present in the body in these cases, in the form of soluble salts, (a supposition which, for lead especially, is very doubtful,) and many remedies founded on chemical views had for their object to form insoluble compounds with the poisonous substance, such compounds being thought to have no action on the living economy. The sulphate of lead, which, so far from being without action on the living body, inevitably causes death within a limited period—a fact which leads to a distinction between slow and rapid poisoning.

According to the old chemical view, therefore, the object was to render insoluble a poison which was supposed to be previously soluble. The object of the treatment by the iodide of potassium, on the contrary, is to render soluble metallic compounds that have become fixed in the body, to the end that, being again in a state to be taken up by the blood, they may be cast out of the system.

The iodide may be given in doses of from 30 to 93 grains a day for an adult, without producing any unpleasant consequences. It traverses the system with great rapidity, being found in the urine a few minutes after its administration by the mouth. The kidneys are the principal outlet of the iodide of potassium. It is even with extreme difficulty that this salt can be made to pass through the bowels into the stools. Every mercurial compound which can possibly occur in the living economy, even metallic mercury itself, is soluble in iodide of potassium; the presence of the organic substances of the body does not hinder these reactions.

Let us examine now the facts, on the strength of which I would induce medical men to employ the iodide of potassium in chronic disorders produced by poisonous metallic compounds.

1. M. Boucher, house-painter. Saturnine pains in the spine, (Rachialgie,) incomplete paralysis of the arms. Lead colic treated with success.

From the 10th December to the 13th March, he took 3087 grains of the iodide of potassium, when he was perfectly cured.

2. A type-founder complained of termina, and of weakness of the legs. 2778 grains of the iodide entirely restored him.

3. A workman of forty or fifty years of age, who had worked in a white lead manufactory, had been in hospital more than six weeks. When first subjected to the iodide of potassium treatment he was weak and quite broken up.

It is almost impossible to form an idea of the rapidity of the amendment which ensued in this man. He grew better, so to speak, as you stood by. At the end of three weeks he left the hospital completely cured. The dose of the iodide had been raised pretty rapidly; when he left the hospital he was taking 77 grains a day.

4. A man about fifty years old had been subjected to different modes of treatment without benefit. When he began his treatment he could scarcely hold himself up; all his limbs were more or less palsied; he was pale and emaciated. After five or six weeks' treatment he was perfectly well, and left the hospital at his own request.

5. Ordinarily, dogs and cats die within a short time in the greater number of establishments where lead and its compounds are worked: rats and mice do not harbor there. All animals exhibit symptoms analogous to those which are observed in man, and are benefited by the same treatment.

Neither sulphuric acid, nor sulphates, can serve as antidotes to slow poisoning by the salts or compounds of lead: the sulphate of lead being itself a slow, but sure poison, capable of killing vigorous dogs in 20 or 30 days.

The evidence commonly adduced is not sufficient to prove that sulphuric acid is an antidote to slow lead poisoning, although sulphate of magnesia may be properly given in cases of poisoning by a soluble salt of lead, to act on the portion yet unabsorbed.

Sulphate of lead is, probably, less readily absorbed than other compounds, but that sulphuric acid will reach lead already deposited in the system is quite unproved. Moreover, sulphate of lead is itself a poison, as can be shown by experiments on dogs, some of which are detailed. One dog was paralyzed on the eleventh day, after 108 grains of the pure sulphate was given; he then refused food, and died epileptiform, emaciated, and in a state approaching scurvy, on the 22d. Another dog took 293 grains, and died on the 28th day, and with similar symptoms.

When a great excess of sulphate of lead is administered, the phenomena of lead poisoning are not in ratio to the quantity of poison administered. Experiments on dogs prove this. It appears that only a certain portion can be absorbed—the rest passes with the excrement.

If, to a dog that has been for some time under the poisonous influence of sulphate of lead, iodide of potassium be administered suddenly, in pretty large doses, death will ensue. If, on the contrary, the two drugs be given concurrently, the dog will suffer no harm. Iodide of potassium may be employed, therefore, as a prophylactic.

Experiments on dogs, very carefully made, prove this assertion, and show that the minute quantity of lead which traverses the body disappears, without causing much harm if it meets with a sufficient quantity of iodide of potassium to favor its expulsion. When, on the contrary, lead compounds accumulate in the body of the animal, death is inevitable. For even if the iodide be given, dangerous phenomena ensue more rapidly in consequence of the larger quantity of the poison which is thus rendered active and thrown into the current of the circulation.—*Ibid.*

---

*Iodide of Potassium a remedy for the affections caused by Lead and Mercury.*—We find in the January number of the London edition of the British and Foreign Medico-Chirurgical Review, a very valuable paper under the above head, first published by M. Melsens, in the *Annales de Chimie et de Physique.* By a series of experiments on man and dogs, conducted with great care, with the results accurately noted, M. Melsens has conclusively shown, that the iodide of potassium is not only a safe, certain and radical cure for the common forms of *saturnine* and *mercurial* poisoning, but an equally sure preventive of the injurious effects so frequently produced by emanations from lead and mercury. M. M. assumes, that in all cases of lead and mercurial poisoning the metallic substance is in actual union with the affected part or parts, and is there retained in the form of some *insoluble* compound. He believes that in these instances the iodide of potassium, after it reaches the circulation, combines with the metallic poison, and forms with it a new and *soluble* salt; liberates the poison from its union with the part or parts, dissolves it, and thus suffers it to again enter the circulation. Once in the circulating fluids, (in the form of a double iodide of mercury and potassium,) M. Melsens supposes these metals to be eliminated from the blood and tissues through the kidneys, nearly as soon as formed, in combination with any excess of the iodide that may chance to be present.

M. Melsens details a number of cases of mercurial poisoning, in which the iodide produced a rapid and permanent cure; and in some instances, the elimination of the mercury by the kidneys, was positively ascertained by reagents.

Most of us have had evidences of the anti-mercurial influence of the iodide of potassium upon the human subject; we have seen persons who had been the victims of mercurial poisoning, after taking the potash a short time, become profusely salivated. Does not this fact demonstrate that the potash liberates the mercury from the tissues, throws it into the circulation, and leaves it free to act on the glandular system?

M. Melsens has proven, by repeated experiment, that if to a dog that has been for some time under the poisonous influence of sulphate of lead, iodide of potassium be administered suddenly in pretty large doses, death will ensue. If, on the contrary, the two drugs be given concurrently, the dog will suffer no harm. Iodide of potassium may, therefore, be employed as a prophylactic in lead-poisoning.—*Condensed by Ed. N. O. Med. and Surg. Journal.*

---

*Arsenical Confectionery.*—At an inquest held last week at Ashford, upon two brothers killed by eating the painted ornaments of a twelfth cake, Professor Taylor, who analyzed the stomachs of the deceased children, said that he detected arsenic, which the system had completely absorbed. In his opinion, the yellow color of the ornaments that contained the poison was produced by orpiment, or sulphate of arsenic. The quantity that he discovered did not exceed a quarter of a grain. The green color of these ornaments was imparted by the arsenic of copper, which poisoned in small doses. During the two last years he had met with ten fatal cases from children eating these ornaments.—*Ibid.*

---

"An exchange paper states that sixty students in the medical department of the University of Louisville, Ky., are to be seen every Sabbath morning in the Sabbath School of the Chestnut St. Presbyterian Church, diligently engaged in the study of the Holy Scriptures, under the instruction of Drs. Yandell and Silliman, two of the University Professors."

To our mind, the above simple announcement speaks volumes in favor of the young gentlemen referred to. We shall fear nothing for the science of medicine when it is in the hands of competent men, of established christian principles. As a rule, it is not they who are "carried about by every wind" of medical doctrine.—*New Jersey Med. Reporter.*

